# Clinical traits and systemic risks of familial diabetes mellitus according to age of onset and quantity: an analysis of data from the community-based KoGES cohort study

**DOI:** 10.4178/epih.e2023029

**Published:** 2023-02-23

**Authors:** Ju-Yeun Lee, Kyungsik Kim, Sangjun Lee, Woo Ju An, Sue K. Park

**Affiliations:** 1 Department of Preventive Medicine, Seoul National University College of Medicine, Seoul, Korea; 2 Integrated Major in Innovative Medical Science, Seoul National University College of Medicine, Seoul, Korea; 3 Department of Ophthalmology, Myongji Hospital, Hanyang University College of Medicine, Goyang, Korea; 4 Cancer Research Institute, Seoul National University, Seoul, Korea; 5 Department of Biomedicine Sciences, Seoul National University Graduate School, Seoul, Korea

**Keywords:** Diabetes mellitus, Family, Age of onset, Cohort studies, Cardiometabolic risk factors

## Abstract

**OBJECTIVES:**

The aim of this study was to clarify the clinical trait of familial diabetes mellitus (DM) by analyzing participants’ risk of DM according to the age of DM onset in parents and siblings, and to evaluate individuals’ risk of DM-associated cardiometabolic diseases.

**METHODS:**

Altogether, 211,173 participants aged ≥40 years from the Korean Genome and Epidemiology Study were included in this study. The participants were divided into groups based on the number (1 or 2 relatives) and age of onset (no DM and early, common, or late onset) of familial DM. Participants’ risk of DM was assessed using a Cox regression model with hazard ratios and 95% confidence intervals (CIs). A logistic regression model with odds ratios was used to evaluate associations among the participants’ likelihood of acquiring cardiometabolic diseases such as hypertension, chronic kidney disease (CKD), and cardiovascular disease.

**RESULTS:**

The risk of developing DM was 2.02-fold (95% CI, 1.88 to 2.18) and 2.88-fold (95% CI, 2.50 to 3.33) higher, respectively, in participants with 1 and 2 family members diagnosed with familial DM. It was 2.72-fold (95% CI, 2.03 to 3.66) higher in those with early-onset familial DM. In the early-onset group, the respective risks of hypertension and CKD were 1.87-fold (95% CI, 1.37 to 2.55) and 4.31-fold (95% CI, 2.55 to 7.27) higher than in the control group.

**CONCLUSIONS:**

The risk of DM and related cardiometabolic diseases was positively associated with the number of family members diagnosed with DM and an early diagnosis in family members with DM.

## GRAPHICAL ABSTRACT

[Fig f1-epih-45-e2023029]

## INTRODUCTION

Over the past few decades, the prevalence of diabetes mellitus (DM) has steadily increased worldwide. Although there is a globally agreed-upon goal of halting this dramatic increase in DM, approximately 422 million people worldwide at all income levels have DM. Since DM is a chronic condition that can lead to serious damage to the blood vessels, heart, nerves, eyes, and kidneys, the prevention and management of DM and its possible complications are important in public health.

Genetic predisposition is a risk factor for many chronic diseases, including DM, and the manifestation of a genetic predisposition is mediated by several environmental factors [1]. Given that DM has complex traits, its onset may occur earlier or later in life, reflecting hereditary or environmental influences, respectively. Family history is an important risk factor for the development of DM, both as a genetic effect and as a lifestyle effect of health behavior or eating habits [2]. Family history is relevant because it is a strong and easily assessed risk factor [3]; however, the implications of the timing of DM onset in family members are understood less well. If 1 family member has early-onset DM, individuals are more likely to be genetically disposed to DM, but they may also be more concerned with preventing or managing the disease. Thus, it would be valuable to investigate the individual risk of DM according to the age of DM onset in an individual’s family members.

In a report on DM in the Korean population, approximately 14% of adults aged ≥ 30 years had DM in 2014, and nearly a quarter of Korean adults had pre-DM [4,5]. Among them, 30% of all persons with DM were unaware of their condition, and 11% of people with DM remained untreated. In addition, nearly 30% of individuals with DM had albuminuria or impaired renal function [4]. Therefore, there has been an increasing emphasis on early identification and appropriate management for people at risk of developing DM in Korea.

In light of these important findings, we focused on the clinical trait of family history in DM patients and aimed to investigate individuals’ risk of developing DM according to the ages of DM onset among their siblings and parents. We also assessed individuals’ specific risks of potentially DM-consequent cardiometabolic diseases, according to the clinical traits of familial DM. This may be of practical value in screening programs and may improve our understanding of the clinical role of familial DM, ultimately helping to reduce the burden of cardiometabolic diseases.

## MATERIALS AND METHODS

### Data sources

Data from participants aged ≥ 40 years in the community-based Korean Genome and Epidemiology Study (KoGES), a cohort study conducted from 2001 to 2016 in Korea, were used to investigate the heritability of DM according to familial DM status and the effect of DM on cardiometabolic diseases. The KoGES data were established to serve as a research platform containing a health database and biobank for investigating genetic and environmental etiologies of diseases in Koreans [6]. We used the following 3 population-based study datasets: the Ansan and Ansung study cohort, the Health Examinees study cohort, and the Cardiovascular Disease Association Study cohort. Detailed information has been presented in a previous study [6].

### Study population and definition of main risk factors

All study participants were enrolled upon their first visit. The presence of DM was assessed in each participant during the first and follow-up visits. To establish reliable cohort data, the time between the first and follow-up visits was set similarly for each dataset. Only participants who completed their follow-up visits were included in the final analysis. For each participant, we censored the follow-up time at the index date of the follow-up visit.

The presence of DM in the parents and siblings of each participant was assessed during the first visit as well. To capture a relatively specific measure of the main risk factors, we categorized parental DM status as (1) both parents free of DM; (2) at least 1 parent with early-onset DM (e.g., if 1 parent had early-onset and the other had late-onset DM, they were classified as early-onset DM); (3) 1 or both parents with common-onset DM (e.g., if 1 parent had common-onset and the other had late-onset DM, they were classified as common-onset DM); and (4) 1 or both parents with only late-onset DM. These 4 categories were mutually exclusive. Family histories of early-onset, common-onset, and late-onset DM were defined as individuals for whom the age of the first diagnosis of DM in family members was as follows: < 45 years, 45-59 years, and ≥ 60 years, respectively [7]. We also classified parental DM as unilateral (1 parent with DM) or bilateral (both parents with DM). We categorized the DM status of siblings and first-degree and second-degree relatives into the same groups as parental DM status.

### Definition of outcome variables

DM was defined as the primary outcome variable by combining the questionnaire and the blood test results. For our study, participants diagnosed with DM according to the questionnaire or those with glycated hemoglobin of ≥ 6.5%, fasting plasma glucose level of ≥ 126 mg/dL, or plasma glucose level of ≥ 200 mg/dL 2 hours after an oral glucose tolerance test were considered to have DM. This definition was also applied to identify subsequent DM cases during the follow-up period.

As a secondary outcome variable, information on cardiometabolic diseases was collected. Diagnostic histories of cardiometabolic diseases were assessed to determine whether a family history of DM was related to the likelihood of other cardiometabolic diseases. The cardiometabolic diseases investigated in this study were hypertension (HTN), chronic kidney disease (CKD), and cardiovascular disease (CVD), including myocardial infarction, angina, coronary artery disease, stroke, cerebral hemorrhage, and other cerebral artery diseases. CKD was defined as having a glomerular filtration rate (GFR) of < 60 mL/min per 1.73 m2. GFR was estimated using the CKD Epidemiology Collaboration (CKD-EPI) estimation equation, using data on sex, age, and serum creatinine. The diagnostic histories of cardiometabolic diseases other than CKD were collected from the questionnaire.

### Statistical analysis

We compared the basic demographic characteristics of the study groups using the Pearson chi-square test for categorical variables and analysis of variance for continuous variables. For the primary analysis, a Cox proportional-hazards regression model was used to estimate the risk of each family history condition for DM on the development of new DM cases relative to non-DM participants in the cohort data. The risk of developing DM was evaluated using the hazard ratio (HR).

For the secondary analysis, we performed logistic regression analysis to estimate the odds ratio (OR) of each cardiometabolic disease in each study group. Since the study period was too short to detect the development of cardiometabolic diseases, there would be few incident cases of these diseases. Because the prevalence of cardiometabolic diseases during the study period was low, we assumed that the OR approached the relative risk based on the raredisease assumption [8]. Thus, the concurrent risk of cardiometabolic diseases was evaluated using ORs. We selected factors as covariates in the multiple models that were considered as risk factors for DM and correlated with a family history of DM (yes vs. no), but were not mediators between the family history of DM and the development of DM and had no collinearity among the independent variables (examined using the Pearson correlation coefficient and the variance inflation factor). The selected covariates were age, sex, alcohol intake (abstinent, low to medium, or high), smoking status (current, former, or non-smoker), body mass index (BMI), waist-hip ratio, systolic blood pressure, total and high-density lipoprotein (HDL) cholesterol, fasting glucose, alanine aminotransferase, γ-glutamyl transpeptidase, and active physical activity (yes or no). Each of these covariates was adjusted using multiple Cox regression and logistic regression models. All analyses were performed using the SAS version 9.4 (SAS Institute Inc., Cary, NC, USA). Statistical significance was set at p-value < 0.05. The results are presented as mean± standard deviation.

### Ethics statement

Institutional review board approval by Seoul National University Hospital (IRB No. 2012-105-1183) was obtained. The review board requirement for obtaining patients’ written informed consent was waived because all personal identifying information was removed from the dataset prior to analysis. This study adhered to the principles stipulated in the Declaration of Helsinki.

## RESULTS

### Demographics

A total of 211,173 participants in the established data were enrolled and categorized by parental or sibling age at DM onset. After participants with a history of DM at baseline were excluded, the analysis included a final total of 203,303 participants. The groups differed in terms of fasting glucose level, HbA1c, and energy intake, and more noticeably in terms of smoking and drinking habits (Table 1). The mean follow-up duration was 61.3± 20.4 months (range, 20-145).

### Risk of diabetes mellitus (DM) development among participants according to the age of onset of familial DM

In the unadjusted analyses, having at least 1 parent with DM increased the study participants’ risk of DM, compared to no parental DM (p< 0.001). Adjustment for covariates did not alter the results. In the multivariate analysis, having 1 parent (unilateral) and both parents (bilateral) with DM significantly increased the participants’ risk of DM (HR, 2.03; 95% confidence interval [CI], 1.87 to 2.21; and HR, 2.69; 95% CI, 2.00 to 3.61, respectively), as compared to the control group (Table 2). Having at least 1 parent with common-onset DM was associated with a higher risk of DM (HR, 2.50; 95% CI, 2.19 to 2.87; p< 0.001).

In the sibling DM group, a trend similar to the parental group was observed. Having at least 1 sibling with DM increased the risk of DM as compared to having no siblings with DM (p< 0.001). Having at least 1 sibling with early-onset DM showed a higher risk in participants as well (HR, 2.61; 95% CI, 1.87 to 3.64; p< 0.001).

In the entire first- and second-degree relative group, having more relatives with DM was associated with a higher risk in participants of developing DM (p< 0.001). A remarkably higher risk of DM was found in the group with 2 or more familial DM cases (HR, 2.88; 95% CI, 2.50 to 3.33) and in the group with early-onset familial DM (HR, 2.72; 95% CI, 2.03 to 3.66).

### Cardiometabolic diseases among participants according to the age of onset of familial diabetes mellitus

Participants’ ORs for cardiometabolic diseases, including HTN, CKD, and CVD, are presented in Table 3 by study group. In participants with DM, familial DM was significantly associated with higher ORs for HTN, CKD, and CVD than having no familial DM (all p< 0.001). The ORs of HTN, CKD, and CVD were significantly higher in the DM participants with both parental and sibling DM than in the control group (OR, 1.81, 2.82, and 1.71, respectively). In the group with early-onset parental and sibling DM, the OR of HTN was 1.87-fold (95% CI, 1.37 to 2.55) higher, and that of CKD was 4.31-fold (95% CI, 2.55 to 7.27) higher, than in the control group. In the early-onset group, the OR of HTN in female participants was 2.50-fold (95% CI 1.66 to 3.77) higher and the OR of CKD in male participants was 5.80-fold (95% CI, 2.97 to 11.34) higher (Supplementary Material 1). At the time of HTN, CKD, and CVD diagnoses in both sexes, the early-onset group was younger than the control group (Supplementary Material 2).

## DISCUSSION

In the present study, we estimated the relative risks of DM development and related cardiometabolic diseases among participants according to their familial DM status. Considering that the participants included in the cohort were ≥ 40 years of age, it is very likely that the incidental cases were type 2 DM. In the primary analysis, we observed that the participants’ risk of developing DM was 2.02-fold and 2.88-fold higher in those with 1 and 2 family members with familial DM, respectively. It was 2.72-fold higher in the group with early-onset familial DM. In the secondary analysis, the likelihood of developing HTN, CKD, and CVD showed a tendency to increase significantly in participants with early-onset familial DM and a larger number of family members with DM. Notably, in the early-onset group, female participants’ likelihood of having HTN was 2.50-fold higher, and male participants’ likelihood of having CKD was 5.80-fold higher.

It has been widely reported that a family history of DM is a useful risk factor for DM and related diseases in offspring. Alharithy et al. [9] reported that having a parental history of type 2 DM was significantly correlated with diagnosis at a younger age. Other researchers reported that parental history of type 2 DM was associated with a later onset of type 1 DM in offspring [10]. However, most previous studies focused only on the effect of family history on individuals with DM [7-11], and data on the consequences of parental and sibling DM according to age at onset are still scarce.

In our primary findings for DM development, we demonstrated that early-onset DM and a larger number of relatives with DM were highly predictive factors of participants’ type 2 DM. This clinical finding can be explained by a combination of genetic and environmental traits. Age at onset has been demonstrated as an indicator of genetic susceptibility in many chronic diseases, including DM. In previous studies, a stronger genetic susceptibility was shown to be associated with an earlier onset of type 1 DM and a greater risk in first-degree relatives [12,13]. Another study found that the cumulative incidence of DM in offspring increased in parallel with a decrease in age at onset of DM in the fathers [14]. We also showed a similar transmission pattern of type 2 DM. The genetic predisposition of family members is also important in type 1 and 2 DM. In addition to genetic traits, most lifestyle factors, including smoking, drinking, and low physical activity, as well as fasting glucose and HbA1c levels, were significantly higher in the early-onset group than in the other groups. This supports the observation that among family members, shared lifestyle factors that contribute to DM are associated with an accelerated onset of DM [15,16]. Our findings allow clinicians to give appropriate advice to individuals about the possible development of DM, depending on the age of onset of parental or sibling DM.

In the secondary findings of related cardiometabolic diseases, we found that early-onset familial DM also led to a higher likelihood and earlier diagnosis of associated cardiometabolic diseases in each participant. More HTN cases at an earlier age were found in females with early-onset familial DM. By contrast, more CKD cases at an earlier age were found in males with early-onset familial DM. This indicates another significant role of genetic traits in familial DM regarding the development of cardiometabolic diseases among the participants. Because various risk factors for metabolic syndrome commonly coexist, family members with DM may have other risk factors [17]. This aggregated tendency may affect participants equally. As a result of the possibility of aggregated risk factors for metabolic syndrome, the association between cardiometabolic diseases may be significant. It is unclear whether the association is attributable to a familial DM history or the participants’ own DM status. Based on our results, although the participant’s own DM had a great influence, it seems that familial DM, particularly sibling DM history, may also have an effect on the likelihood of cardiometabolic outcomes in participants without DM. Further studies are required to verify this possibility.

In contrast to the fact that CKD had a stronger association in the participants with early-onset familial DM, CVD showed a relatively lower or insignificant association in each study group. This may be due to differences in the natural history of CKD and CVD, the late diagnosis of CVD following CKD, an insufficient number of outcomes due to the early-onset group’s small size, and an insufficient follow-up period for CVD development. Thus, careful interpretation is needed when applying the findings, and further well-controlled clinical studies are required to verify these results.

This study has several limitations. Because the original KoGES data were based on the recall of retrospective information, participants with qualifying medical statuses may have been undercounted. However, since participants were not aware of the main purpose of this study, there was a lower chance of recall bias. For younger participants, it may not have been possible to assess the age of onset comprehensively for family members’ DM. Because of the short follow-up period, it may not have been possible to determine for some participants whether a family member had DM. However, the current study focused on “early onset” within the traceable follow-up range. Moreover, significant results with similar tendencies toward increased risk were observed despite the potential insufficiency of recruitment. Therefore, the current results may underestimate the association due to dilution of the original results, but this possibility is not expected to fundamentally change our conclusions. Further long-term follow-up studies are required to confirm this hypothesis. A major strength of the current study is its large sample size, which provided sufficient statistical power and prospective potential. Our data highlight the clinical trait of a family history of DM, highlighting the risk of early-onset familial DM for the development of type 2 DM and related cardiometabolic diseases among study participants. In addition, the present study assessed study participants’ risk of developing various cardiometabolic diseases, classified according to sex and details of the family members’ DM status.

In conclusion, this analysis of data from a Korean-specific cohort study presents evidence for several issues that were not appropriately demonstrated in earlier studies conducted in other populations. The current study is distinctive in that it focused on the effects of age of onset in family members as well as the impact of a family history of DM, which is a known risk factor. In particular, we highlighted the effects and risks of early-onset familial DM. We demonstrated that participants’ risk of DM and cardiometabolic diseases increased as the number of family members diagnosed with DM increased, and this relationship was especially prominent for participants who had a family member with early-onset DM. Based on our results, family history has significant genetic and environmental impacts on the occurrence of DM, suggesting that the risk of DM may vary depending on the DM status of family members. This information could be used effectively for managing DM and its complications in individuals with a family history of DM. Further studies are required to confirm these findings.

## Figures and Tables

**Figure f1-epih-45-e2023029:**
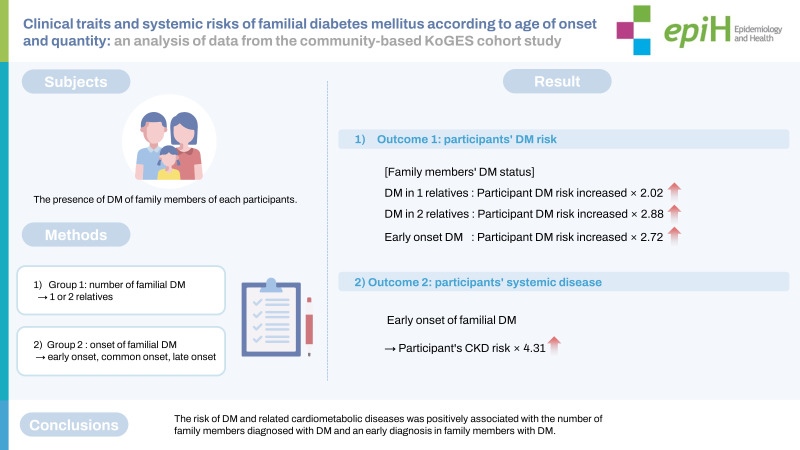


**Table 1.** Selected characteristics of study groups at baseline from the Korean Genome and Epidemiology Study

DM status of relatives	No DM	Late-onset DM	Common-onset DM	Early-onset DM	p-value^1^	DM in 1 relative	DM in ≥2 relatives	p-value^1^
Total participants (n)	176,685	13,203	12,025	1,390		30,206	4,682	
Male	64,124 (36.3)	4,223 (32.0)	3,672 (30.5)	449 (32.3)	<0.001	9,401 (31.1)	1,315 (28.1)	<0.001
Low income (≤3 decile)	56,591 (40.1)	3,292 (28.6)	3,044 (28.3)	396 (33.5)	<0.001	7,674 (30.3)	1,211 (30.7)	<0.001
Current smokers	74,795 (42.3)	697 (5.3)	545 (4.5)	110 (7.9)	<0.001	1,832 (6.1)	227 (4.9)	<0.001
Current drinkers	78,243 (44.3)	6,100 (46.2)	5,586 (46.5)	667 (48.0)	<0.001	13,896 (46.0)	1,980 (42.3)	<0.001
Low physical activity	89,337 (50.5)	6,026 (45.6)	5,323 (44.3)	682 (49.1)	<0.001	14,113 (46.7)	2,118 (45.2)	<0.001
Age (yr)	54±9	52±8	51±8	50±7	<0.001	52±8	53±8	<0.001
Body mass index (kg/m^2^)	23.9±2.9	23.9±2.9	24.1±3.0	24.1±3.0	<0.001	24.0±2.9	24.2±3.0	<0.001
Systolic blood pressure (mmHg)	123.3±16.2	121.4±15.2	121.9±15.2	121.1±15.8	<0.001	121.9±15.4	122.3±15.1	<0.001
Diastolic blood pressure (mmHg)	77.0±10.4	75.7±10.0	75.8±10.0	76.2±10.5	<0.001	76.1±10.1	75.9±9.8	<0.001
Serum total cholesterol (mg/dL)	232±1,854	205±869	213±1,287	269±2,677	0.206	211±1,148	240±2,060	0.141
Energy intake per day (kcal)	1,753±599	1,773±594	1,764±600	1,795±611	<0.001	1,771±595	1,725±578	<0.001
Fasting plasma glucose (mg/dL)	95±20	98±25	100±29	102±31	<0.001	100±27	106±33	<0.001
HbA1c (%)	5.7±0.7	5.8±0.9	5.9±1.0	6.1±1.3	<0.001	5.8±0.9	6.2±1.2	<0.001

Values are presented as number (%) or mean±standard deviation. DM, diabetes; HbA1c, hemoglobin A1c.

1 p-values for differences among the 3 groups: tested with analysis of variance for continuous scales or chi-square test for categorical scales.

**Table 2.** Association between a family history of diabetes and the development of participants' own diabetes among cohort members in the Korean Genome and Epidemiology Study

Group		Incident DM cases (n)	Non-DM cases (n)	HR (95% CI)^1^	HR (95% CI)^2^
No parental diabetes		4,527	68,712	1.00 (reference)	1.00 (reference)
At least 1 parent with diabetes					
	Unilateral parental diabetes	884	7,841	2.20 (2.04, 2.37)	2.03 (1.87, 2.21)
	Bilateral parental diabetes	57	388	3.25 (2.50, 4.23)	2.69 (2.00, 3.61)
No parental diabetes		4,527	68,712	1.00 (reference)	1.00 (reference)
At least 1 parent with diabetes					
	Late onset	429	4,379	1.99 (1.81, 2.21)	1.87 (0.99, 3.40)
	Common onset	260	2,206	2.90 (2.60, 3.29)	2.50 (2.19, 2.87)
	Early onset	14	152	2.31 (1.37, 3.91)	1.83 (0.98, 3.40)
No sibling diabetes		4,860	72,901	1.00 (reference)	1.00 (reference)
At least 1 sibling with diabetes					
	1 sibling	514	3,628	2.05 (1.87, 2.24)	1.90 (1.71, 2.11)
	≥2 siblings	94	412	3.71 (3.02, 4.54)	2.74 (2.17, 3.47)
No sibling diabetes		4,860	72,901	1.00 (reference)	1.00 (reference)
At least 1 sibling with diabetes					
	Late onset	95	683	1.91 (1.56, 2.34)	1.68 (1.34, 2.12)
	Common onset	288	2,057	2.37 (2.11, 2.67)	2.09 (1.83, 2.38)
	Early onset	49	338	2.44 (1.84, 3.23)	2.61 (1.87, 3.64)
No. of diabetes in first- to second-degree relatives					
	0	4,057	65,447	1.00 (reference)	1.00 (reference)
	1	1152	10,075	2.18 (2.04, 2.32)	2.02 (1.88, 2.18)
	≥2	259	1,419	3.45 (3.04, 3.91)	2.88 (2.50, 3.33)
No relative with diabetes		4,057	65,447	1.00 (reference)	1.00 (reference)
Diabetes onset in first- to second-degree relatives					
	Late onset	470	4,702	2.03 (1.84, 2.23)	1.87 (1.68, 2.08)
	Common onset	519	4,058	2.69 (2.45, 3.06)	2.45 (2.21, 2.71)
	Early onset	62	479	2.88 (2.09, 3.45)	2.72 (2.03, 3.66)

DM, diabetes mellitus; HR, hazard ratio; CI, confidence interval.

1 Adjusted for age and sex.

2 Adjusted for age, sex, cigarette smoking, alcohol consumption, physical activity, total energy intake, body mass index, waist-hip ratio, systolic blood pressure, and blood levels of total cholesterol, high-density lipoprotein cholesterol, triglyceride, alanine aminotransferase, pulse rate, γ-glutamyl transpeptidase, and fasting glucose.

**Table 3.** Associations between a family history of diabetes and the likelihood of participants' own systemic diseases at baseline from Korean Genome and Epidemiology Study

Status of family history for DM			HTN cases, n (%)	OR (95% CI)^1^	CKD cases, n (%)	OR (95% CI)^2^	CVD cases, n (%)	OR (95% CI)^2^
Parental DM	Sibling DM	Participant DM						
-	-	-	36,252 (21.3)	1.00 (reference)	6,569 (3.9)	1.00 (reference)	7,719 (4.5)	1.00 (reference)
-	-	+	7,333 (43.5)	1.95 (1.87, 2.02)	1,723 (10.3)	2.09 (1.94, 2.25)	1,647 (9.8)	1.55 (1.45, 1.65)
+	-	+	1,423 (37.5)	2.06 (1.91, 2.22)	247 (6.5)	2.64 (2.26, 3.08)	274 (7.2)	1.62 (1.40, 1.85)
-	-	-	37,079 (20.5)	1.00 (reference)	6,467 (3.6)	1.00 (reference)	7,734 (4.3)	1.00 (reference)
-	-	+	7,589 (42.5)	2.01 (1.93, 2.09)	1,661 (9.3)	2.09 (1.94, 2.25)	1,654 (9.3)	1.56 (1.46, 1.66)
-	+	+	1,167 (41.8)	1.81 (1.66, 1.98)	309 (11.1)	2.82 (2.42, 3.28)	267 (9.6)	1.71 (1.48, 1.98)
-	-	-	34,127 (21.1)	1.00 (reference)	6,185 (3.8)	1.00 (reference)	7,247 (4.5)	1.00 (reference)
-	-	+	6,403 (43.5)	1.98 (1.89, 2.06)	1,459 (9.9)	2.00 (1.85, 2.16)	1,440 (9.8)	1.55 (1.45, 1.67)
+	+	+	2,353 (40.7)	2.00 (1.87, 2.12)	511 (8.6)	2.74 (2.44, 3.09)	481 (8.1)	1.63 (1.47, 1.82)
-	-	-	34,127 (21.1)	1.00 (reference)	6,185 (3.8)	1.00 (reference)	7,247 (4.5)	1.00 (reference)
+/ Late	+/ Late	+	759 (34.3)	1.91 (1.71, 2.12)	160 (8.3)	2.61 (2.14, 3.18)	157 (8.1)	1.54 (1.28, 1.86)
+/ Common	+/ Common	+	850 (38.2)	1.91 (1.74, 2.11)	153 (6.9)	2.60 (2.15, 3.15)	171 (7.7)	1.69 (1.43, 2.00)
+/ Early	+/ Early	+	99 (39.3)	1.87 (1.37, 2.55)	23 (8.0)	4.31 (2.55, 7.27)	13 (4.5)	1.03 (0.52, 2.03)
-	-	-	34,127 (21.1)	1.00 (reference)	6,185 (3.8)	1.00 (reference)	7,247 (4.5)	1.00 (reference)
-	+	-	2,125 (26.6)	1.13 (1.07, 1.20)	384 (4.8)	1.14 (0.99, 1.32)	472 (4.5)	1.20 (1.07, 1.35)
+	-	-	2,952 (15.4)	1.00 (0.96, 1.05)	282 (1.5)	0.95 (0.82, 1.10)	487 (2.6)	0.99 (0.89, 1.09)
+	+	-	339 (18.3)	0.99 (0.85, 1.12)	39 (2.1)	1.00 (0.67, 1.47)	74 (4.0)	1.09 (0.82, 1.44)

DM, diabetes mellitus; HTN, hypertension; CKD, chronic kidney disease; CVD, cardiovascular disease; OR, odds ratio; CI, confidence interval.

1 Adjusted for age, sex, cigarette smoking, alcohol consumption, physical activity, body mass index, and blood levels of total cholesterol, total energy intake, and fasting glucose.

2 Adjusted for age, sex, cigarette smoking, alcohol consumption, physical activity, body mass index, systolic blood pressure, and blood levels of total cholesterol, total energy intake, and fasting glucose.

## SUPPLEMENTARY MATERIALS

Supplementary Material 1. Association with the familial history of diabetes and the likelihood of participants' own systemic diseases according to the sex

Supplementary Material 2. Association with the familial history of diabetes and age of diagnosis of participants' own systemic diseases according to the sex
