# Supplementary material for: Clinical traits and systemic risks of familial diabetes mellitus according to age of onset and quantity: an analysis of data from the community-based KoGES cohort study
Source: Epidemiol Health. 2023 Feb 23;45:e2023029. doi: 10.4178/epih.e2023029 (PMC10586928; doi:10.4178/epih.e2023029)
Supplement: Supplementary Material 1. — Association with the familial history of diabetes and the likelihood of participants' own systemic diseases according to the sex [file epih-45-e2023029-Supplementary-1.docx]

**Supplementary Material 1. Association with the familial history of diabetes and the likelihood of participants' own systemic diseases according to the sex**

| **Status of family history for DM** | | | **HTN cases**  **N (%)** | **OR (95% CI)^1^** | **CKD cases**  **N (%)** | **OR (95% CI)^22^** | **CVD cases**  **N (%)** | **OR (95% CI)^2^** |
| --- | --- | --- | --- | --- | --- | --- | --- | --- |
| **Men** | | |  |  |  |  |  |  |
| Parental DM | Sibling DM | Subject DM |  |  |  |  |  |  |
| - | - | - | 13815 (23.5) | 1.00 | 2690 (4.6) | 1.00 | 3587 (6.1) | 1.00 |
| - | - | + | 3332 (41.2) | 1.73 (1.63-1.83) | 784 (9.7) | 1.86 (1.67-2.06) | 903 (11.2) | 1.51 (1.38-1.66) |
| + | - | + | 630 (36.4) | 1.83 (1.63-2.05) | 119 (6.9) | 2.57 (2.07-3.19) | 145 (8.4) | 1.50 (1.24-1.83) |
|  |  |  |  |  |  |  |  |  |
| - | - | - | 14312 (22.9) | 1.00 | 2673 (4.3) | 1.00 | 3632 (5.9) | 1.00 |
| - | - | + | 3569 (40.6) | 1.80 (1.71-1.90) | 781 (8.9) | 1.90 (1.72-2.11) | 932 (10.6) | 1.53 (1.40-1.67) |
| - | + | + | 393 (37.8) | 1.45 (1.25-1.68) | 122 (11.7) | 2.62 (2.06-3.34) | 116 (11.2) | 1.57 (1.26-1.96) |
|  |  |  |  |  |  |  |  |  |
| - | - | - | 13241 (23.3) | 1.00 | 2560 (4.5) | 1.00 | 3405 (6.0) | 1.00 |
| - | - | + | 3020 (41.3) | 1.77 (1.67-1.88) | 684 (9.4) | 1.81 (1.62-2.02) | 812 (11.1) | 1.53 (1.39-1.68) |
| + | + | + | 942 (37.5) | 1.72 (1.56-1.89) | 216 (8.7) | 2.57 (2.16-3.06) | 236 (9.4) | 1.55 (1.33-1.82) |
|  |  |  |  |  |  |  |  |  |
| - | - | - | 13241 (23.3) | 1.00 | 2560 (4.5) | 1.00 | 3405 (6.0) | 1.00 |
| +/ Late | +/ Late | + | 336 (38.1) | 1.72 (1.47-2.02) | 71 (8.1) | 2.25 (1.67-3.03) | 87 (9.9) | 1.54 (1.19-1.99) |
| +/ Common | +/ Common | + | 340 (36.7) | 1.66 (1.43-1.93) | 72 (7.8) | 2.65 (2.01-3.49) | 79 (8.5) | 1.44 (1.12-1.85) |
| +/ Early | +/ Early | + | 42 (30.4) | 1.29 (0.80-2.09) | 16 (11.6) | 5.80 (2.97-11.34) | 9 (6.5) | 1.03 (0.41-2.57) |
| **Women** | | |  |  |  |  |  |  |
| Parental DM | Sibling DM | Subject DM |  |  |  |  |  |  |
| - | - | - | 22437 (20.2) | 1.00 | 3879 (3.5) | 1.00 | 4132 (3.7) | 1.00 |
| - | - | + | 4001 (45.6) | 2.15 (2.04-2.27) | 939 (10.7) | 2.31 (2.08-2.56) | 744 (8.5) | 1.57 (1.43-1.74) |
| + | - | + | 793 (38.3) | 2.22 (2.00-2.47) | 128 (6.2) | 2.65 (2.13-3.31) | 129 (6.2) | 1.70 (1.39-2.08) |
|  |  |  |  |  |  |  |  |  |
| - | - | - | 22767 (19.2) | 1.00 | 3794 (3.2) | 1.00 | 4102 (3.5) | 1.00 |
| - | - | + | 4020 (44.2) | 2.21 (2.09-2.33) | 880 (9.7) | 2.27 (2.04-2.53) | 722 (7.9) | 1.58 (1.43-1.73) |
| - | + | + | 774 (44.3) | 2.03 (1.81-2.27) | 187 (10.7) | 2.90 (2.38-3.54) | 151 (8.6) | 1.80 (1.49-2.18) |
|  |  |  |  |  |  |  |  |  |
| - | - | - | 20886 (19.9) | 1.00 | 3625 (3.5) | 1.00 | 3842 (3.7) | 1.00 |
| - | - | + | 3383 (45.6) | 2.19 (2.06-2.32) | 775 (10.5) | 2.19 (1.95-2.45) | 628 (8.5) | 1.58 (1.42-1.76) |
| + | + | + | 1411 (41.3) | 2.17 (1.99-2.35) | 292 (8.5) | 2.84 (2.42-3.32) | 245 (7.2) | 1.69 (1.45-1.96) |
|  |  |  |  |  |  |  |  |  |
| - | - | - | 20886 (19.9) | 1.00 | 3625 (3.5) | 1.00 | 3842 (3.7) | 1.00 |
| +/ Late | +/ Late | + | 423 (40.1) | 2.01 (1.74-2.31) | 89 (8.5) | 2.87 (2.19-3.78) | 70 (6.7) | 1.50 (1.14-1.97) |
| +/ Common | +/ Common | + | 510 (39.2) | 2.09 (1.84-2.38) | 81 (6.2) | 2.51 (1.92-3.29) | 92 (7.1) | 1.91 (1.53-2.40) |
| +/ Early | +/ Early | + | 57 (37.8) | 2.50 (1.66-3.77) | 7 (4.6) | 2.99 (1.26-7.06) | 4 (2.7) | 1.01 (0.37-2.78) |

Abbreviation: DM, diabetes; HTN, hypertension; CKD, chronic kidney disease; CVD, cardiovascular disease; N, number; OR, odds ratio; CI, confidence interval

Adjusted for age, sex, cigarette smoking, alcohol consumption, physical activity, body mass index, blood levels of total cholesterol, total energy intake, and fasting glucose

2. Adjusted for age, sex, cigarette smoking, alcohol consumption, physical activity, body mass index, systolic blood pressure, blood levels of total cholesterol, total energy intake, and fasting glucose
